# *‘We know what the barriers are, but nothing changes’* - Professional perspectives on promoting breast cancer screening initiatives for ethnic minority women

**DOI:** 10.1186/s12913-026-14705-w

**Published:** 2026-06-04

**Authors:** Helen Morley, Omolade Allen, Gillian Hutchison, Victoria G. Woof, David P. French

**Affiliations:** 1 Manchester Centre of Health Psychology, Division of Psychology and Mental Health, School of Health Sciences, University of Manchester, Coupland Street, Manchester, M13 9PL England; 2 NIHR Manchester Biomedical Research Centre, Manchester Academic Health Science Centre, Central Manchester University Hospitals NHS Foundation Trust, Manchester, England; 3 Division of Medical Education, School of Medical Sciences, University of Manchester, Manchester, M13 9PL England; 4 Nightingale Breast Cancer Centre, Wythenshawe Hospital, Manchester University NHS Foundation Trust, Manchester, M23 9LT England

**Keywords:** Breast cancer, Breast cancer screening, Health inequalities, Ethnic minority women, Professional views, Qualitative research, Interviews, Minoritised communities

## Abstract

**Background:**

Despite long-standing efforts to increase breast cancer screening uptake, there are substantial inequalities in access and outcomes for ethnic minority women. Despite much research focussing on the views of minoritised groups on barriers to their attendance, systemic factors have received limited attention.

**Methods:**

Seventeen professionals responsible for implementing initiatives to promote screening equity for minoritised groups took part in semi-structured online interviews. Data were analysed using reflexive thematic analysis.

**Results:**

Three themes were developed: (1) Structural inequities: moving from system centred design to community centred care: professionals described entrenched systemic misalignments that disadvantage women from minoritised communities, (2) Hostile environment effects on healthcare engagement: wider societal racism and exclusionary policies were seen to undermine trust and access, (3) Innovation within constraints: participants described grassroots, community centred strategies, though these often relied on individual initiative rather than system level support.

**Conclusions:**

Professionals working to promote breast screening uptake for ethnic minority women described numerous systemic challenges that hinder equity. Structural reform to breast screening programmes which goes beyond translation and cultural competency is needed to produce greater equity in uptake for minoritised communities. Findings highlight the importance of designing healthcare systems so that they are more appropriate for ethnic minority women.

**Supplementary Information:**

The online version contains supplementary material available at 10.1186/s12913-026-14705-w.

## Introduction

Breast cancer is the most diagnosed cancer among women globally and remains a leading cause of mortality in the UK, where one in seven women will be diagnosed during their lifetime [1–3]. Since its introduction almost four decades ago, the NHS Breast Screening Programme (NHSBSP) has significantly improved early detection and survival. Recent statistics, suggest approximately 98% of women diagnosed with stage 1 breast cancer survive at least five years compared with fewer than three in ten diagnosed at stage 4 [4].

Despite these successes, participation in screening remains unequal. Currently, the UK national average for screening uptake is 70% which is in fact the national target [5, 6]. However, attendance can vary across and within regions with noticeably lower attendance in ethnically diverse and socioeconomically deprived areas. For example, in Lancashire, Northwest England, where there is a large British Pakistani population, uptake among eligible women is 54–59%, considerably below the national average [5, 7, 8]. These disparities mean that women from minoritised communities are more likely to be diagnosed at later stages, contributing to poorer outcomes and perpetuating health inequalities [9–12].

A substantial body of previous research has identified numerous barriers to screening participation. These include fear of diagnosis, fatalistic beliefs, embarrassment, cultural taboos around cancer, language barriers, and low awareness of breast health [10, 13–20]. Additional practical challenges such as lack of transport, childcare, and appointments scheduling further hinder attendance. In response, interventions such as GP endorsement, reminder texts or calls, and targeted health promotion have been tested [21–24]. Whilst some, like GP endorsement demonstrate promise, many others show inconsistent or limited effectiveness [25–27].

A likely explanation for these mixed results lies in the challenges of implementation. Interventions are often developed without fully considering realities faced by the professionals responsible for delivering them, including limited resources, competing demands, and the need for additional training [28–30]. Despite much research on the perspectives of patients on the barriers to attendance, frontline professional views are often absent from the research literature on improving screening uptake. Professionals working within breast screening services, primary care, and community outreach are uniquely placed to understand these barriers and to identify practical, culturally responsive approaches that may succeed where standard strategies have struggled [15, 31, 32]. These professionals can provide informed comment on structural issues within healthcare systems that contribute to inequalities in breast screening, such as issues related to resource allocation, training, and organisational culture [25, 33–35]. By understanding these systemic challenges, the NHSBSP can work towards implementing structural changes that support healthcare professionals and providers in delivering equitable and accessible breast screening services to women from minoritised communities [36].

The present study aims to describe the perspectives and experiences of professionals actively working on initiatives to address these inequalities; to inform the development and implementation of novel culturally responsive strategies to improve breast cancer screening uptake for women from minoritised communities.

## Methods

### Design

The study adopted a qualitative cross-sectional design using one-to-one semi-structured online interviews with professionals whose roles involved implementing breast cancer screening initiatives for women from minoritised communities.

### Participants and recruitment

Participants were eligible if they were aged 18 or above; and were involved in implementing initiatives for promoting breast screening uptake among women from minoritised communities in the Greater Manchester region in northern England. Screening uptake varies widely, with some areas considerably below the national average with numerous initiatives to the address these differences [37].

Greater Manchester has a total population of approximately 2.5 million people and includes some of the most deprived areas in England. It is also one of the most ethnically diverse populations in England [38, 39]. Manchester continues to lead regional and contributes to national efforts to address health inequalities. This is underpinned by its commitment to the Marmot’s principle (becoming a Marmot City Region), which advocates action on the social determinants of health, ensuring the best start in life, fair employment and income, healthy and sustainable communities, and a strong focus on prevention across the life span [40, 41].

Data collection took place between April 2024 and June 2025. Participants were recruited using purposive sampling through locally established professional networks including NHS, public health, and third-sector organisations, with snowball sampling used to extend recruitment via colleagues working in related roles. Initial invitations were circulated via local networks and third-sector partners, with study information shared through poster advertisements and email updates distributed across relevant teams. Recipients were encouraged to forward the invitation to others engaged in breast screening or health inequalities work. Regular contact was maintained with key contacts who agreed to support recruitment and with individuals who expressed interest in participating.

In total, 32 individuals were approached or identified through these routes. Of these, six were not eligible because their roles did not involve breast screening initiatives, and three did not respond despite follow-up email requests. The remaining 23 expressed interest, of whom 17 met the inclusion criteria and agreed to participate in an interview. Inclusion criteria required participants to have professional experience in designing, coordinating, or delivering initiatives aimed at improving breast screening uptake among women from minoritised communities.

In line with the principles of reflexive thematic analysis [42, 43] the notion of information power [44] was used to guide and justify the sample size. The study had a focussed aim and drew on a highly specific sample of professionals with direct experience of implementing breast cancer screening initiatives for women from minoritised communities. The research was informed by a critical realist perspective, seeking to understand how structural conditions and professional practices shape service delivery. Interviews generated detailed accounts of implementation experiences, and reflexive thematic analysis was used to interpret patterns of meaning across the dataset. Sample sufficiency was assessed iteratively during data collection and early analysis. Recruitment ended when additional interviews were no longer contributing new insights relevant to the study aims.

### Procedure

Participants were given the option to complete the interview either online or in-person. All participants opted for an online interview which was conducted via Microsoft Teams. Participants provided written consent ahead of the interview. Transcriptions were transcribed via an external third-party organisation, checked and amended by HM.

The interview topic guide (see supplementary file) was developed by HM with input from the research team. Relevant discussions regarding initiatives with colleagues within Greater Manchester also informed its development. The interviews explored participant perspectives on implementing breast cancer screening initiatives for women from minoritised communities, including which approaches were perceived to be effective and why, as well as the challenges and facilitators encountered by professionals in addressing screening inequalities. Participants also had the opportunity to ask any questions at the end of the interview. Feedback sought from the first two interviews regarding the topic guide confirmed that it addressed pertinent topics and was thorough. The topic guide was not changed and was used flexibly during participant interviews.

### Data analysis

Data were analysed using reflexive thematic analysis [42, 43], informed by a critical realist perspective [45]. A critical realist approach aligns with the study’s aim to generate explanations that are not only practically useful for professionals but also attentive to the broader determinants of health inequalities [46]. NVivo was used for data management. The analysis was predominantly inductive, exploratory, and data-driven [47]. HM conducted an initial close reading of all transcripts to support familiarisation with the dataset and generated initial codes, attending to both semantic content (participants’ explicit accounts) and latent meanings (underlying assumptions and structural processes relevant to the research question). Codes were then examined and interpreted to develop candidate themes. These were subsequently reviewed and refined through collaborative discussions with three other members of the research team (DPF, VGW, and OA). Consistent with a reflexive thematic analysis approach, these discussions were used to deepen interpretation and analytic insight rather than to seek coding agreement. Through iterative engagement with the data, themes were developed, reviewed, and defined. Themes were critically examined and revisited in relation to the original data to ensure coherence and resonance with participant narratives.

### Reflexivity, positionality and rigour

The researchers reflected on the fact that four of the team were female, and one was male. The author of this study is a woman who was born and socialised in working class contexts in Manchester. She has experience of working in the NHS in physical and mental health settings, both clinically and research based. The rest of the research team have clinical and research expertise in breast cancer screening, health inequalities research, and qualitative methodology in applied healthcare research. In keeping with reflexive thematic analysis, member checking was not conducted, as the analysis sought to generate an interpretive understanding of shared meanings rather than to validate findings against individual participant accounts. Analytical rigour was supported through iterative engagement with the data, reflexive team discussions, and ongoing attention to the coherence and grounding of themes.

## Results

Seventeen professionals whose role entailed promoting breast screening uptake in women from minoritised communities were interviewed. Participants came from a range of NHS, public health, and voluntary-sector roles, including managers, clinicians, community engagement staff, and charity professionals. Their work included both planning and coordinating initiatives and delivering activities on the ground, with some participants working across both areas. Sixteen participants worked within Greater Manchester, and one worked in an adjacent urban area of Cheshire. Participants were primarily female (*n* = 15) with a mean age of 47.0 years (SD = 8.2; *n* = 16). One participant chose not to disclose their age. Years of experienced ranged between two and over 30 years, with a mean of 16.8 years (SD = 9.6; *n* = 17). Interviews lasted between 24 and 75 min (mean = 52 min) and followed a semi-structured format, allowing flexibility to explore issues most relevant to each participant’s role and experience. While all interviews addressed the study aims, not every section of the topic guide was covered in equal depth in each interview. Shorter interviews focussed on specific areas of implementation aligned with participants’ responsibilities. Further participant characteristics are detailed in Table 1.

**Table 1:** Participants professional role and self-identified ethnicity

Role	Participants (*n* = 17)
*Cancer screening improvement leads (CSILs)	4
NHS Manager	3
Health inequalities lead/ Manager	2
Charity programme manager	1
Charity CEO	1
Health inequalities and engagement officer	1
Public health cancer prevention manager	1
Screening and immunisation coordinator	1
Clinical service manager	1
Research assistant	1
Medical professional/ Surgeon	1
**Ethnicity**	**Participants (n = 17)**
White British	10
British Pakistani	3
Asian Other	1
Black African	1
South African/ Italian/ British	1
White Jewish	1

*This role involves coordinating local screening improvement work/ programmes, liaising across primary care/screening services/public health, and supporting targeted initiatives

Three themes were developed: (1) *Structural inequities: moving from system centred design to community centred care* (2) *Hostile environment effects on healthcare engagement* and (3) *Innovation within constraints* that illuminate both the challenges and opportunities for improving breast screening equity. Participant quotations were selected to reflect perspectives across the range of professional roles included in the study. To preserve anonymity and given the small number of individuals working in some specialised professional roles, job titles were grouped using broader descriptors (e.g. NHS employee or NHS Manger). Additionally, participant interviews focussed on women from communities with lower screening uptake. These include women of South Asian heritage (specifically, Bangladeshi and Pakistani women), and women of Black heritage (Black African and Black Caribbean women).

## Theme 1: Structural inequities: moving from system centred design to community centred care

A fundamental tension voiced by all participants was between the knowledge of what minoritised women need to access breast cancer screening whilst still operating within systems designed around institutional rather than community needs. Rather than isolated service gaps, participants described systematic misalignment that perpetuated instead of addressing health inequities. 

### Structural inflexibility and service misalignment

Participants expressed frustration with systemic inaction despite decades of evidence about barriers to screening uptake with one commenting *“we know what the barriers are, but nothing changes” (NHS Manager)*. This was not simply about resource constraints but about fundamental service design that prioritises institutional convenience over accessibility. Participants saw issues with the outdated “hard to reach” stance. Having previously placed the emphasis on the individual this has now shifted with to focus on the service design:

*I think the challenges are more around the services, rather than the people. So, you know, we call these people hard to reach, but I feel that the services are hard to reach. And especially with the Breast Screening Service, the system is so old and culturally they’ve been doing things in a certain way. (NHS employee)*

This mirrors a broader shift moving towards system centred language (e.g. ethnic minority to minoritised communities). Despite knowing that invitation letters require high health literacy levels and that translated materials exist but remain inaccessible, these barriers persist unchanged. The maze of contacts women may encounter when seeking language support illustrates multiple points of possible failure:

*So, for me, I think the biggest one is like, they’ll get a letter in English, if they can’t read the English, it says, if you need it in a different language ring this number. Then, even if they can read that bit, they ring, and there’s another person on the phone, saying, what can I do? And they can’t speak the language. (NHS employee)*

These examples illustrate how apparently neutral administrative decisions systematically disadvantage specific communities, revealing the unintended consequences of seemingly objective service delivery models. Locations for the mobile breast screening van revealed another aspect of structural misalignment. Vans are often situated in areas suitable for the unit (i.e. with access to electricity points) but not for some women whose journeys to these locations would be highly inconvenient. While participants could identify areas of greatest need (those characterised by high deprivation and ethnic diversity) often the inflexibility of NHSBSP further demonstrated how systems neglect to accommodate community realities. Although there is a possibility that women may access other screening opportunities elsewhere. As one participant explained:

*But some of the ones that are specifically lower, like especially in the Rochdale area, they had a high South Asian cohort of patients and I found that out through speaking to the practice manager […] The reason why they have a high DNA [did not attend] rate is because their screening round was in winter, because they have six months to attend, but a lot of their clients go abroad for the winter. (NHS employee)*

Participants often questioned the lack of structural adaptations in the NHSBSP set-up, and whether it was fit for purpose for women from varied ethnic minority groups. This insight highlights the conscientiousness of participants working in breast screening services. Professionals are keen for services to be appropriate and adapted for the different communities they are serving.

### The cycle of invisibility

Participants felt that without meaningful demographic data, healthcare systems continue to overlook women from minoritised communities, reinforcing a cycle of invisibility. This cycle of invisibility was understood to operate through both passive and active mechanisms. Passive mechanisms relate to system level failures to collect adequate data, meaning some women are not fully represented. Active mechanisms arise when women choose to disengage from services they perceive as unwelcoming or unsafe, for example because of concerns about how their immigration status may be handled.

Participants articulated how data paucity makes evidence-based advocacy more difficult. The aggregation of ethnicity data also masked important within group differences. Pooling of data can obscure variations in attendance between groups (e.g. Black African women and Black Caribbean women) potentially leading to an inaccurate understanding of these two different populations. This can affect the development of targeted and effective interventions. This was a source of frustration with participants recognising the potential harm this approach can take and limit:

*I think the big challenge we’ve had specifically with black women is that there is no data, and then if there is data it’s, like, for Nigerian black women and it’s almost like everyone’s been grouped together, which is really quite concerning and wrong. (Senior charity employee)*

However, without data, women remain unseen in the system:

*Well again I think it’s the data and the records, you know, so what are those additional needs, is there a language that’s better for you to understand the information in? […] I think we need to be better at recording preferences and needs adjustments and things like that. (NHS Manager)*

Participants also described challenges in how ethnicity information was recorded and used within existing recording procedures. Although this study did not examine how these data were collected in practice, it relates to the recording and usability of ethnicity data within existing administrative systems. These data are typically collected as part of standard NHS demographic recording, though the present study did not evaluate the procedures used locally. One participant described how, after introducing an ethnicity data collection initiative in spring 2025, staff appeared to use discretion in whether to ask this question:

*We ran a report recently about whether ethnicity was actually being asked by our staff and it did become apparent that they were only asking the white women because it was like a simple question to ask so it does look like there’s some avoidance. (NHS Manager)*

This selective implementation reflects broader issues about cultural sensitivities, and the need for staff training that goes beyond procedural compliance to address underlying discomfort with discussing ethnicity and cultural difference.

Where more comprehensive demographic data were available, participants described how this enabled more targeted and responsive interventions. Participants highlighted that challenges arose both from older, fragmented data systems and from inconsistent practices in collecting and recording information such as ethnicity or preferred language. Recent investment within the Greater Manchester Health and Social Care Partnership has included a major overhaul of data infrastructure, replacing disparate spreadsheets with an integrated Microsoft SQL data warehouse and Tableau as part of a wider digital strategy [48]. Participants explained that these developments allowed services to bring together routinely recorded demographic information for the first time, supporting collaboration across organisations. By enabling analysis of variables such as ethnicity and language preference where these had been recorded, the system helped identify specific patterns of inequality. For example, a decline in breast screening coverage among some South Asian women, allowing for more tailored service responses rather than relying solely on general population messaging.

## Theme 2: Hostile environment effects on healthcare engagement

This theme captures how broader societal factors including immigration policy, institutional racism, and xenophobia intersect to shape women from minoritised communities’ engagement with healthcare services. Participants recognised that healthcare delivery occurs within a wider ‘hostile environment’ that affects trust, safety perceptions, and willingness to engage with public services. The broader impact of the ‘hostile environment’ can be significant for women from minoritised communities. Resulting in delayed presentation, thus later stage diagnosis, contributing to poorer outcomes and perpetuating health inequalities.

### Discriminatory experiences and differential treatment

The broader ‘hostile environment’ was explicitly recognised as affecting healthcare engagement.

*The other issues are about racism, and the current hostile environment makes a lot of communities feel very marginalised, if not downright unwanted. So those are big barriers to come through. (NHS employee)*

Participants acknowledged institutional racism within healthcare systems and described witnessing differential treatment based on appearance, language, or cultural markers. These were not isolated incidents but part of broader patterns that undermined trust and discouraged healthcare engagement. Several participants described assumptions made about language preferences based on names, with materials automatically sent in languages other than English without consultation:

*Sometimes I think we make it harder by saying, oh well, hang on, everybody who’s got an Asian name should be sent a leaflet in an Urdu language. We’re second and third generation over here now, so Urdu or Punjabi or Hindi, it’s not going to be their first language, English is their first language. (NHS employee)*

These practices reflect what one participant termed ‘pre-conceived ideas’ about communities that limit rather than enhance accessibility. Another participant commented on these assumptions:

*So I feel that sometimes when we go into these communities, we have a pre-conceived idea of that community, and we kind of expect them to behave this way or react like this, and we’re not open to anything, because we’ve already made those assumptions, and I think a lot of people do those, and I think they shouldn’t stereotype. And I think that’s one of the biggest barriers, going in already feeling like you know that community, especially if you think you’re one of those community (NHS employee)*

However, participants also noted positive cultural shifts within services, particularly with increased workforce diversity. For one breast screening service the change was dramatic. An increase in staff from different countries, and who were younger, had in essence given ‘a new lease of life’ to the team, with patients reporting improved rapport with the mammographers during their appointment. This suggests that diversifying the healthcare workforce and changing organisational culture can help address some hostile environment effects.

### Cultural myths and stigma

Participants identified specific cultural factors that required sensitive, individualised approaches rather than standardised responses. These included cancer stigma and lack of awareness, religious and modesty concerns, family decision-making dynamics, and misconceptions about cancer causation. Importantly, participants recognised the diversity within ethnic minority communities and the dangers of stereotypical assumptions.

Modesty concerns were frequently mentioned: *We don’t show that area, we don’t talk about that area. To all of a sudden to have to come and plonk yourself half naked onto this machine (NHS employee)*

However, participants emphasised that responses needed to be individualised rather than based on ethnic assumptions.

### Theme 3: Innovation within constraints

Despite operating within resource constrained and structurally inflexible systems, participants demonstrated tenacious creativity in developing locally responsive solutions often challenging the status quo. These innovations emerged from insight, community knowledge, and relationship-building rather than policy directives or adequate resource allocation.

### Community-centred alternatives

Despite systemic constraints, participants had extensive knowledge about effective approaches that prioritised community partnership and cultural responsiveness. These approaches recognised community diversity, prioritised trust-building, and positioned community members as partners rather than passive recipients of services. The concept of ‘community-conduits’ was particularly powerful; respected community members who could bridge healthcare systems and communities. Cancer champions represented a successful example of this approach:

*Our cancer champions are a huge part of that because cancer champions are a social movement, yeah. So, they’re not volunteers, yeah. (Senior charity employee)*

Faith leader engagement demonstrated how community-centred approaches leverage existing trust networks:

*So, the pastor from that church, that pastor says that, okay, it’s important that women, you know, you need to go for screening. After giving out the message and then reminds women to go for screening, then they will take it so seriously because it’s from their pastor. (University employee)*

These approaches succeeded because they recognised the social embeddedness of health decisions and worked within existing community structures rather than expecting communities to adapt to healthcare system requirements. This analysis moves beyond individual behaviour change models to recognise how structural inequalities shape health choices, informing more appropriate intervention strategies.

### Grassroots adaptations and workarounds

Participants developed practical workarounds that demonstrated both their commitment to equity and their detailed understanding of community needs. These innovations were characterised by local responsiveness arising from frontline experience rather than top-down planning. Targeted follow-up calls to women who had not attended appointments was one example of a grassroots approach. Participants described how they used their knowledge of local communities to identify women who might benefit from support in a preferred language and organised multilingual staff to make follow-up calls:

*There are 433 patients that were DNAs (did not attend) in April. We went off the names and obviously they don’t speak in English as a first language. I think there were about 40 that she [admin support] ended up doing in total, but she got like 27 people to rebook, which is more than 50 per cent. (NHS employee)*

Participants described this as a small, practical adaptation that helped address barriers they encountered in everyday practice, rather than relying only on the standardised invitation processes. Similarly, participants developed a simple, yet practical, solution to address modesty concerns at screening appointments by offering hospital gowns and ensuring women knew these were available.

The concept of ‘making every contact count’ led to innovative community engagement strategies. Rather than depend on formal health promotion materials, participants embedded screening messages in everyday community interactions:

*People can go out and to this job really well. So GM cancer had some funding for every GM locality, and it was £20,000. What they wanted us to use it on was comms. I thought about it and it and I thought can’t we have people rather than Facebook posts and things. (Senior local government employee)*

This approach recognised that personal interaction and trusted relationships were more effective than ‘behind the desk’ communication methods.

### Digital solutions and equity considerations

Participants identified the possibilities of technology, while remaining aware of the digital divide that could exacerbate rather than reduce health inequalities. This reflected understanding of how apparently universal solutions could have differential impacts.

Digital innovations included QR codes linking to multilingual video resources: *QR codes now, isn’t it, so you know, people scan a QR code, they can go to our website, and they can watch a video around breast screening (NHS employee)*

The digital debate is layered. Participants were aware that some women may experience digital exclusion and might not have the ability to use iPads, computers, or online resources to manage their healthcare. Moreover, due to digital poverty women may not be able to fully participate in online life due to lack of essential resources including internet access, devices, or basic digital literacy:

*All of the stuff is available online, to download, but again, you’ve got to have access to do that, you know, we know that there’s digital poverty, we know that there’s… barriers to that, as well.” (NHS Manager)*

Instead, participants suggested multilayered approaches that combined digital innovations with alternative access methods and digital literacy support. They recognised that the upcoming digital transition (planned for introduction in 2026 via the NHS app and other digital tools) required careful equity considerations to avoid widening existing health inequalities. Furthermore, there might be an opportunity to support and plan interventions with women who will be reaching screening age in the next five to ten years and how health professionals, providers and the systems support women through the digital transition.

## Discussion

Our findings highlight both the constraints and creative potential described by participants within current healthcare systems, offering insight into how more community centred approaches to breast cancer screening services might be developed. They also emphasise the importance of individualised assessment rather than ethnic profiling and the role of cultural humility in service delivery.

Our findings suggest that inequalities in screening participation may be sustained not only by individual level barriers but also by what participants described as a cycle of invisibility operating through both passive and active mechanisms. Passive mechanisms relate to limitations in how demographic data are captured, structured, and used within healthcare systems, which can leave some populations less visible in planning and evaluation processes. Active mechanisms arise when women disengage from services they perceive as unwelcoming, unsafe, or misaligned with their needs, further reducing their visibility within routine data. Together, these processes can constrain the ability of services to recognise and respond to inequalities. Signifying that improving participation requires attention not only to outreach and behaviour change, but also to the structure and relational conditions through which populations become visible to the system. This understanding aligns with wider multi-level models of cancer screening participation, which emphasise the role of organisational structures, service design, and system capacity alongside individual factors [49].

Previous research has focussed on patient level barriers to screening, such as stigma, fear, fatalism, and modesty [14, 19, 25, 50]. By contrast, the present study explored the perspectives of professionals tasked with implementing responses to these challenges. Participants echoed longstanding concerns about cultural stigma, language barriers, and a lack of awareness of breast cancer, or mistrust in healthcare systems, findings that are consistent with previous studies [16, 17, 19, 20, 51]. This is also consistent with evidence highlighting that screening inequalities are shaped by intersecting structural, service-level, and sociocultural factors, including communication practices, accessibility of services, and experiences of discrimination within the healthcare systems [25]. Our findings further illustrate how professionals attempt to navigate these issues in practice, and how systemic constraints such as inflexibility in programme design, limitations in demographic data, and infrastructure challenges can shape efforts to include women from minoritised communities. Similar structural and organisational barriers have been identified in previous research examining inequalities in cancer screening access [52].

Taken together, participants’ accounts suggest the value of complementing individual level interventions with attention to system level conditions that influence equitable uptake. Descriptions of rigid systems and limited demographic data contributed to a perceived cycle of invisibility, and services not always aligned with local needs resonate with wider calls for proportionate universalism and culturally safe care [53–55]. National screening policy has similarly recognised the need for equity focussed approaches, noting that limited granular data and persistent cultural and structural barriers continue to affect participation in some communities [56]. The theme of systemic inflexibility reflects concerns raised in the Marmot Review [53], which highlighted patterns whereby those with the greatest need may receive less effective care. Participants’ accounts also highlight the practical tension inherent in delivering population-based screening programmes within publicly funded systems, where standardised approaches are necessary to operate at scale and within finite resources. This highlights the importance of combining centralised programme structures with locally tailored adaptations to address the needs of specific communities. Participants provided local examples, such as winter screening rounds in some South Asian communities or inaccessible mobile unit locations, as illustrations of how such mismatches can occur in practice.

Participants’ emphasis on community-based approaches, faith and community leadership, and relational trust aligns with socioecological models of health promotion and prevention [57, 58]. This perhaps suggests that these approaches are being implemented locally, even in the absence of formal top-down mandates. Participants described these strategies as ways of responding directly to women’s expressed concerns such as fear, mistrust, or language barriers by working through trusted relationships, culturally familiar settings, and tailored communication rather than relying on standardised invitations. Systematic review evidence further suggests that culturally sensitive outreach, reminders and navigation support can improve screening uptake among underserved populations, highlighting the importance of combining relational and structural strategies [27]. These accounts point to the importance of pairing structural improvements such as better data infrastructure and flexible service models with sustained engagement and trust building within communities if screening programmes are to reduce, rather than repeat, existing inequalities.

### Strengths and limitations

A key strength of this study is its focus on professional perspectives, an under researched area that provides valuable insight into implementation challenges and enablers within the UK healthcare system. The use of reflexive thematic analysis supported depth and flexibility in the analysis, and the principle of information power guided an appropriate sample. Greater Manchester offered a relevant setting for this research given its diverse population and ongoing efforts to reduce health inequalities [38, 40].

Some limitations should be noted. All but one participant was based in Greater Manchester. Therefore, the region’s particular policy context and infrastructure may influence the experiences described which may limit transferability to other settings. Participants were also largely engaged in equity-focussed work, so less supportive views are likely to be underrepresented. Discussions of racism or service shortcomings may have been influenced by social desirability despite assurances of confidentiality. Finally, while the sample included a range of professional roles, it did not include radiographers nor radiologists, whose perspectives may have added further insight.

### Service delivery implications

This study highlights the need for structural reform to address inequalities in breast screening uptake for women from minoritised communities. As reflected in the theme of systemic inflexibility and the perceived cycle of invisibility, participants described how misalignment between service design and community needs could not be resolved through cultural competency training or translated materials alone. Instead, participants emphasised the importance of robust demographic data systems capable of disaggregating screening uptake by ethnicity, language preference, and other relevant characteristics. Accounts of optional application of ethnicity data collection and discomfort in asking about cultural identity further suggest the need for training that goes beyond procedural compliance to address underlying assumptions and confidence in these conversations.

The theme of hostile environment illustrates how wider political and social factors including immigration concerns and experiences of discrimination were understood by participants to shape engagement with screening services. While screening programmes cannot directly address these broader determinants, participants’ accounts indicate the importance of equipping healthcare professionals to recognise how marginalisation influences health behaviours. Trauma-informed approaches, which acknowledge the cumulative effects of exclusion and mistrust, may therefore offer one way of strengthening engagement and trust. Although participants did not explicitly use the term ‘trauma-informed’, their description of fear, avoidance, and mistrust support consideration of such approaches [59, 60].

Participants also highlighted community-based responses, including collaboration with faith leaders, community champions, and locally trusted figures. These examples, from the subtheme of grassroots innovation within constraints, suggest that socially embedded models of engagement can complement formal service delivery. Initiatives such as community conduits or cancer champions align with participants’ emphasis on relational trust and locally tailored outreach.

Finally, participants raised concerns about infrastructure limitations and digital transitions within screening services. In light of the planned digital transformation of the NHS breast screening programme our findings suggest the importance of ensuring that improved infrastructure does not inadvertently widen inequalities [61]. Although digital exclusion was not the primary focus of interviews, participants’ concerns about language barriers, accessibility, and data systems indicate that attention to digital literacy, accessible appointment systems, and inclusive design will be important to prevent further marginalisation. Therefore, embedding equity into infrastructure planning and policy development is likely to be a significant component of future service reform.

### Future research

Future research should prioritise the rigorous evaluation of community-based innovations. While participants demonstrated strong practice based knowledge of what works to engage women from minoritised communities in breast screening, there is little (if any) formal evaluation of these which is needed to establish effectiveness and inform scale-up across different settings [20, 25–27]. As the NHSBSP moves toward digitalisation in 2026, research should also examine how digital tools can be implemented in ways that promote, rather than hinder, equitable access [61]. This includes understanding and addressing barriers related to digital literacy, language, and access to technology, particularly for women at higher risk of exclusion. Additionally, more research informed by theories of intersectionality is needed to explore how women from minoritised communities’ identities (such as social position [class], ethnicity, age, gender) shape help-seeking, screening behaviour and engagement [62].

## Conclusion

This study highlights persistent gaps in breast screening uptake reflect not only individual level barriers but also structural and relational factors within healthcare systems. Limitations in demographic data, inflexible programme design, and misalignment between services and community contexts may contribute to the cycle of invisibility. While locally developed (and often creative) adaptations illustrate the potential for more responsive community-informed approaches. Improving screening equity may therefore require attention not only to awareness raising, but also to how services identify, engage with, and respond to the needs of the local populations they serve. While grounded in one regional context, this study offers insight into how aligning infrastructure, workforce practice, and community engagement may support more equitable access to screening.

## Supplementary Information

Below is the link to the electronic supplementary material.

Supplementary Material 1

## Data Availability

Data is available upon reasonable request from the first author.

## Abbreviations

DNA: Did not attend
Microsoft SQL data warehouse: Microsoft Structured Query Language Data Warehouse
NHSBSP: National Health Service Breast Screening Programme
