# Supplementary material for: ‘We know what the barriers are, but nothing changes’ - Professional perspectives on promoting breast cancer screening initiatives for ethnic minority women
Source: BMC Health Serv Res. 2026 Jun 4;26:1086. doi: 10.1186/s12913-026-14705-w (PMC13459212; doi:10.1186/s12913-026-14705-w)
Supplement: Supplementary file 1 [file 12913_2026_14705_MOESM1_ESM.docx]

**Topic Guide/ Interview Questions**

Topic Guide:  Experiences of promoting Breast Cancer Screening Among Ethnic Minority Women: A Qualitative Study

**Introduction:**
- Introduce the purpose of the interview: to gain insights into the experiences of healthcare providers/ professionals in implementing initiatives for breast cancer screening for ethnic minority women.
- Explain the confidentiality of the interview and how the information will be used.
- Obtain consent for recording the interview either written/ verbal

**Section 1: Professional Background and Experience**
**1. Role and Experience**
- Can you provide an overview of your role and experience in delivering breast cancer screening services?
- What led you to focus on improving breast cancer screening for ethnic minority women?
- Which specific groups do you work with - for example, Black African, Black Caribbean, South Asian women, or everyone?

**Section 2: Implementation of Improvement Initiatives**
**3. Initiatives and Strategies**
- What improvement initiatives have you been involved in to address disparities in breast cancer screening for ethnic minority women?

- Are there any other strategies?
- Can you describe any successes you have experienced in implementing these initiatives?

- Why did this work?

- Would you improve anything?

- Can you describe any challenges you have experienced in implementing these initiatives?

- How/ did you overcome these?

- What would you do differently?

- Are these the “best” kinds of initiatives/ interventions that should be done?

- Is there anything better?

- Are there any other approaches you would like to try/ are planning on trying?

- Can you describe these approaches?

**4. Community Engagement**
- How have you engaged with different ethnic minority women to understand their specific needs and challenges related to breast cancer screening?

e.g., community groups/ faith groups

e.g., religion, access, transport

- How could this have been done better?

- Are there things you should not be doing?

**Section 3: Barriers and Facilitators**
**5. Barriers**
- What are the key barriers you have encountered in delivering effective breast cancer screening services to ethnic minority women?
- Any specific barriers related to language, culture, or socioeconomic factors?

- Prompt: where a person lives? Where a person was born?

**6. Facilitators**
- What do you think helps to improve the uptake of breast cancer screening among ethnic minority women?
- How have you addressed language and cultural barriers in the delivery of breast cancer screening services?

- Can you give me some examples of these?

- Did they work?

- What didn’t work?

**Section 4: Systemic Challenges and Support
7. Systemic Challenges**
- In your opinion, what systemic challenges exist within healthcare systems that contribute to health inequalities in breast cancer screening for ethnic minority women?
**8. Support and Resources**
- What support or resources do you believe are necessary to facilitate the successful implementation of improvement initiatives for breast cancer screening in ethnic minority communities?
- How can healthcare organisations such as the NHS better support healthcare professionals in addressing health inequalities in breast cancer screening?

**Section 5: Policy and Future Directions**
**9. Policy Changes**
- From your perspective, what policy changes or guidelines would better support the delivery of breast cancer screening services to ethnic minority women?
**10. Future Directions**
- What do you envision as the future direction for improving breast cancer screening for ethnic minority women in the UK?
- If you were in charge of the NHS what would you do to address disparities in breast cancer screening for ethnic minority women?

**11. Specific Experiences**
- Can you share any specific experiences or challenges you have encountered when working with ethnic minority women in the context of breast cancer screening?

- How does working in this area make you feel?

- In a good way/ bad way?

**Anything else?**

**12.** Is there anything you else I haven’t asked you today, that you think is important?

**Demographic data collection**

Collect data on age, gender, professional occupation, range of bandings, ethnicity, years’ experience

**Conclusion:**
- Thank the participant for their time and valuable insights.
- Reiterate the confidentiality of the information shared.
- Offer the participant an opportunity to ask any questions or provide additional comments.
- Thank the participant and conclude the interview.
